# Effects of knockdown of miR-210 in combination with ionizing radiation on human hepatoma xenograft in nude mice

**DOI:** 10.1186/1748-717X-8-102

**Published:** 2013-04-25

**Authors:** Wei Yang, Jing Wei, Ting Sun, Fenju Liu

**Affiliations:** 1 Department of Radiobiology, School of Radiological Medicine and Protection, Soochow University, No. 199 Renai Road, Suzhou 215123, China; 2 Brain and Nerve Research Laboratory, The First Affiliated Hospital, Soochow University, No. 708 Renmin Road, Suzhou 215006, China

**Keywords:** MiR-210, Hypoxia, Radiotherapy, Hepatoma

## Abstract

**Background:**

Solid tumors usually develop local hypoxia, which renders them resilient to radiotherapy. MiR-210 is the most consistently and robustly induced miRNA under hypoxia and functions as a micro-controller of a wide range of cellular responses to hypoxia. Hence, it is important to investigate the effect of knockdown of miR-210 in tumorigenesis and evaluate the efficacy of knockdown of miR-210 in combination with radiotherapy on human tumor xenograft in nude mice.

**Materials and methods:**

SMMC-7721 Cells with stable integration of the anti-sense miR-210 were generated through lentiviral-mediated gene transfer and were subcutaneously implanted into nude mice. Mice were monitored for tumor growth and survival after radiotherapy. MiR-210 expression in tumor tissues was assessed by real-time Reverse transcription-Polymerase Chain Reaction (RT-PCR). Protein expression of HIF-1α and miR-210 targeted genes in human hepatoma xenograft was assessed by Western blot. Tumors were analyzed for proliferation, apoptosis, and angiogenesis biomarkers by immunohistochemistry staining.

**Results:**

Tumor growth was delayed in miR-210 downregulated xenograft. Knockdown of miR-210 increased protein expression of miR-210 targeted genes, but decreased HIF-1α protein in hepatoma xenograft. Knockdown of miR-210 in combination with radiotherapy is more effective than radiotherapy alone or miR-210 knockdown therapy alone in suppressing tumor growth and extending survival duration. Combined therapy decreased Ki-67-positive cells and CD31-positive cells and increased TUNEL-positive cells in tumor xenograft.

**Conclusions:**

Knockdown of miR-210 in combination with radiotherapy showed an enhanced anti-tumor effect on human hepatoma xenograft. Our experiments demonstrated specific inhibition of miR-210 expression might be a means to enhance the effectiveness of radiotherapy to human hepatoma.

## Background

Hepatocellular carcinoma (HCC) is the second leading cause of cancer-related deaths worldwide [1]. There are approximately 750,000 new cases of liver cancer, in which 85-90% are HCC, reported globally per year and most of the patients who develop HCC die of it [2]. The treatment of patients with HCC is particularly challenging because of high recurrence rate after surgical resection and resistance to chemotherapy and radiotherapy [3]. As the current therapeutic options for HCC patients are limited, there is an essential need to analyze the molecular oncogenic mechanisms in order to determine novel targets for specific systemic therapy.

HCC tumors, like many other human solid tumors, usually develop local hypoxia, which promotes HCC progression by facilitating angiogenesis and metabolic adaptation and renders them resilient to radiotherapy [4,5]. Adaptation of tumor cells to the hypoxic conditions depends on the hypoxia-inducible factor 1 (HIF-1), a transcriptional activator of cell survival, proliferation, angiogenesis, invasion and metastasis genes [6,7]. Recently, it has been demonstrated that a specific set of microRNAs (miRNAs) molecules are upregulated by hypoxia [8]. MiRNAs are a class of small (21–22 nucleotide in length) single-stranded noncoding RNAs, which participate in crucial biological processes, including development, differentiation, apoptosis, metabolism and tumorigenesis through inhibition of RNA translation or degradation of target messenger RNA (mRNA) by base pairing between their “seed region”, nucleotides 2–8, and their target genes’ 3′ untranslated region (UTR) [9,10]. Among these hypoxia-induced miRNAs, miR-210 is unique in its wide distribution, HIF dependence and robust upregulation in response to hypoxia [11]. Several miR-210 targets which influence cell proliferation, apoptosis, metabolism, and angiogenesis have been identified such as E2F3, MYC antagonist (MNT), caspase-8 associated protein-2 (CASP8AP2), iron-sulfur cluster scaffold protein (ISCU) and the receptor tyrosine kinase ligand ephrin-A3 (EFNA3) [12-16]. Thus, miR-210 functions as a micro-controller of a wide range of cellular responses to hypoxia.

In preliminary studies we employed lentiviral-mediated anti-sense miR-210 gene transfer technique to downregulate miR-210 expression in human hepatoma cells and found that knockdown of miR-210 expression significantly suppressed cell viability, induced cell arrest, increased apoptotic rate and enhanced radiosensitivity in hypoxia [17]. We hypothesis that miR-210 might be a logical novel target to overcome hypoxia-induced radioresistance and knock-down of miR-210 might enhance radiosensitivity of hypoxic cells in hepatoma xenograft through inhibiting proliferation and angiogenesis and inducing apoptosis. In the present study, we investigated the effect of knockdown of miR-210 in tumorigenesis and the efficacy of knockdown of miR-210 in combination with radiotherapy in nude mice bearing human hepatoma SMMC-7721 cells and its mechanism.

## Methods

### Cell line and cell culture

The 293T and human hepatocarcinoma cell line SMMC-7721 were purchased from the Type Culture Collection of the Chinese Academy of Sciences and maintained in Dulbecco’s modified Eagle’s medium supplemented with 10% fetal bovine serum, 100 U/mL penicillin and 100 mg/mL streptomycin, in a 37°C incubator in a 5% CO_2_ humidified atmosphere.

### Generation of stable cell lines

SMMC-7721 Cells with stable integration of the anti-sense miR-210 (5′-TCAGCCGCTGTCACACGCACAG-3′) or scramble sequence (5′-TTCTCCGAACGTGTCACGTTTC-3′) were generated through lentiviral-mediated gene transfer [17]. To generate the respective viruses, 293T cells were transfected with the lentiviral vector, pGLV-anti-210-GFP or pGLV-scr-GFP, along with the packaging plasmid PG-P1-VSVG, PG-P2-REV and PG-P3-RRE using calcium phosphate following standard protocols. The target human hepatocarcinoma SMMC-7721 cells were infected with both of the viruses (encoding either anti-sense miR-210 or scramble sequence) and selected using puromycin. Clonal cell populations carrying anti-sense miR-210 or scramble sequence were obtained by limiting dilution of 100–300 cells in three 96-well plates. After 4 weeks, single clones were analyzed for positive GFP signals. The positive clones were expanded for animal experiments.

### Tumor-bearing mice model and treatment

For *in vivo* implantation, SMMC-7721, SMMC/Lv-scr and SMMC/Lv-anti-210 cells were washed in Hanks’ balanced salt solution (HBSS) and injected subcutaneously at 1 × 10^6^ cells in 0.1 ml HBSS in the right hind limb of 6–8-week-old female Balb/c nude mice (Experimental Animals Center of Shanghai Institute of Life Science, Shanghai, China), respectively. When the diameter of tumor reached about 6 ~ 8 mm, the mice implanted with SMMC-7721 cells (14 days after inoculation) were taken as control and the mice implanted with SMMC/Lv-scr (14 days after inoculation) or SMMC/Lv-anti-210 (21 days after inoculation) cells were randomly divided. The mice implanted with SMMC/Lv-scr cells were divided into two groups: The negative control vector group received no X-irradiation; Radiotherapy group was subjected to 8 Gy X-ray irradiation (6 MV, the dose rate was 100 cGy/min) by a PRIMUS accelerator (SIEMENS Medical Solutions, Erlangen, Germany) at room temperature. The mice implanted with SMMC/Lv-anti-210 cells were divided into two groups: Anti-sense miR-210 therapy group received no X-irradiation; Combined therapy group was subjected to 8 Gy X-ray irradiation. Irradiation was locally confined to the tumors by shielding the rest of the body with lead and was conducted 1 day after dividing. Mice were monitored for tumor growth and survival. All the animal experiments were conducted in accordance with Guidelines for the Welfare of Animals in Experimental Neoplasia and approved by Ethics Committee of Soochow University.

### Real-time reverse transcription-polymerase chain reaction (RT- PCR) analysis of miR-210 expression in tumor tissues

When the diameter of tumor reached about 6 ~ 8 mm, three mice implanted with SMMC-7721 cells, SMMC/Lv-scr and SMMC/Lv-anti-210 cells were killed and the tumors were removed for real-time RT-PCR and Western blot analysis, respectively. Total cellular RNA was isolated from tumor tissue using Trizol reagent (Sangon Inc. Shanghai, China) and transcribed using TaqMan microRNA reverse transcription kit (Applied Biosystems) according to the manufacturer’s protocol. MiR-210 expression was assessed by real-time PCR according to the TaqMan MicroRNA Assay protocol (Applied Biosystems). The 20 μl reactions were incubated in a 96-well optical plate at 95°C for 3 minutes, followed by 40 cycles of 95°C for 12 seconds, and 58°C for 30 seconds. Fold changes in miR-210 expression between treatments and controls were determined by the 2^-ΔΔCT^ method, normalizing the results to U6 RNA expression level.

### Western blot analysis of HIF-1α, MYC antagonist (MNT), ephrin-A3 (EFNA3) and apoptosis-inducing factor, mitochondrion-associated, 3 (AIFM3) protein expression in tumor tissues

Tumor tissues were homogenized in 500 μl sodium chloride-Tris buffer (pH 7.5) containing EDTA and protease inhibitors on ice for 30 s followed by 4 cycles of freezing/thawing. Cell debris was removed by centrifugation at 10,000 g for 10 min at 4°C. Equal amounts of lysate protein were fractionated by sodium dodecylsulfonate (SDS)–polyacrylamide gel electrophoresis at 100 V for 80 min at room temperature. The separated proteins were transferred to a nitrocellulose membrane, which was then probed for 2 h at room temperature with rabbit monoclonal anti-HIF-1α, rabbit monoclonal anti-MNT, rabbit monoclonal anti-EFNA3 and rabbit polyclonal anti-AIFM3 (Santa Cruz Inc., Santa Cruz, CA, USA) and rabbit polyclonal anti-β-actin (Sigma, St Louis, MO, USA). Immune complexes were detected with horseradish peroxidase-conjugated goat antibodies to rabbit immunoglobulin G (Amersham Biosciences, Little Chalfont, England, UK). Immunoblots were visualized by chemiluminescence using a chemiluminescence kit (Invitrogen, Carlsbad, CA, USA) and the specific bands were recorded on X-ray film. Actin protein levels were used as a control to verify equal protein loading.

### Measurement of tumor volume

The tumor growth was monitored by measuring the tumor diameters in two dimensions with a caliper every second day. The tumor volumes were calculated as follows: L(long diameter) × S^2^(short diameter)/2. The formula for tumor inhibition rate is as follows: TIR(*%*) = (1 − [experimental volume/control volume]) × 100.

### Immunohistochemical studies for Ki-67 and cluster of differentiation 31 (CD31) in tumor tissues

The mice used for immunohistochemical studies were sacrificed 1 day after the irradiation. Tumor tissues were fixed and imbedded in paraffin. Tumor sections of 5 μm were cut from the embedded tissue and incubated with specific primary antibodies, including rabbit monoclonal antibody to human Ki-67 (KeyGen Biotech.) and rabbit monoclonal antibody to mouse CD31 (eBioscience, Inc., San Diego, CA, USA) for 1 h at 37°C followed by overnight at 4°C in humidity chamber. Negative controls were incubated only with universal negative control antibodies under identical conditions. The sections were then incubated with appropriate biotinylated secondary antibody for 60 min at room temperature. Thereafter, sections were incubated with conjugated horseradish peroxidase streptavidin (KeyGen Biotech.) for 60 min, followed with 3,3'-diaminobenzidine (Sigma) working solution, and counterstained with hematoxylin. The proliferation index was determined as number of Ki-67-positive (brown) cells/total number of cells × 100, and intratumoral microvessel density (IMVD) was quantified by counting the CD31-positive (brown) cells in 9 most highly vascularized fields (400×) [18,19].

### Detection of apoptotic cells in tumor tissues

Apoptotic cells in tumor tissues were detected by terminal deoxynucleotidyl transferase (TdT)-mediated dUTP-biotin nick end labeling (TUNEL) stain, using an In Situ Cell Death Detection Kit (KeyGen Biotech.) following the manufacturer’s specifications. In brief, tumor histological sections were permeabilized using a mixture containing 0.1% sodium citrate and 0.1% Triton X-100 and incubated with TUNEL reaction mixture containing terminal deoxynucleotidyltransferase and fluorescein-dUTP at 37°C for 60 min. The apoptotic index was calculated as number of apoptotic (brown) cells/total number of cells × 100 in 9 randomly selected fields (400×).

### Statistical analysis

Data are expressed as means ± standard deviations (SD) for separate experiments. Statistical significance was estimated by one-way analysis of variance (ANOVA) followed by a post-hoc Least Significant Difference (LSD) test using the SPSS version 12.0 software. The difference was considered statistically significant when *p* < 0.05.

## Results

### MiR-210 expression in hepatoma xenograft

**Figure 1:**
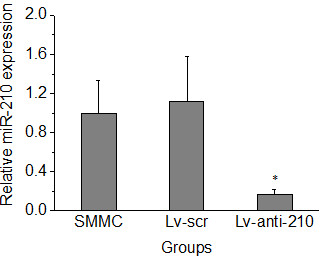
**Real-time Reverse transcription-Polymerase Chain Reaction (RT-PCR) analysis of miR-210 expression (fold change) in human hepatoma xenograft.** Error bars indicate the standard error of the mean of three individual experiments. * indicates *p* < 0.001 vs. SMMC/Lv-scr xenograft.

MiR-210 expression in hepatoma xenograft was quantitatively measured using real-time RT-PCR. MiR-210 expression in SMMC-7721/Lv-anti-210 xenograft was significantly decreased compared with that in SMMC/Lv-scr xenograft (*p* < 0.001). In contrast, no obvious change of miR-210 expression was observed in SMMC-7721/Lv-scr xenograft compared with that in SMMC-7721 xenograft (*p* > 0.05) (Figure 1). These data demonstrate that stable integration of anti-sense miR-210 significantly suppressed miR-210 expression in human hepatoma xenograft in nude mice.

### Effect of knockdown of miR-210 on protein expression of HIF-1α and miR-210 targeted MNT, EFNA3 and AIFM3 genes in hepatoma xenograft

**Figure 2:**
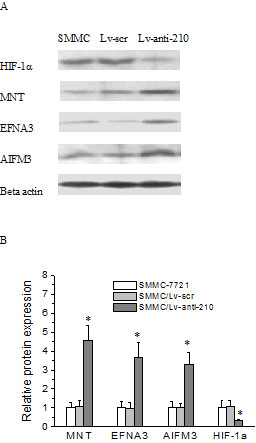
**Detection of protein expression of HIF-1α and miR-210 targeted genes in hepatoma xenograft by Western blot.** The relative levels of protein expression were normalized against protein levels of an internal control gene, β-actin, performed in the same run. (**A**) Western blot of HIF-1α, MNT, EFNA3 and AIFM3 protein. (**B**) Relative levels of HIF-1α, MNT, EFNA3 and AIFM3 protein expression. Error bars indicate the standard error of the mean of three individual experiments. * indicates *p* < 0.001 vs. SMMC/Lv-scr xenograft.

To investigate the mechanism underlying the knockdown of miR-210 mediated growth delay in SMMC-7721/Lv-anti-210 xenograft, we analyzed protein expression of HIF-1α, MNT, EFNA3 and AIFM3 genes in human hepatoma xenograft by Western blot. Experiments were repeated three times. The relative levels of protein expression were normalized against protein levels of an internal control gene, β-actin, performed in the same run. The level of MNT, EFNA3 and AIFM3 protein expression in SMMC-7721/Lv-anti-210 xenograft was significantly increased (*p* < 0.001), while the level of HIF-1α protein expression was significantly decreased (*p* < 0.001), compared with that in SMMC/Lv-scr xenograft. No obvious change of these protein expression was observed in SMMC-7721/Lv-scr xenograft compared with that in SMMC-7721 xenograft (*p* > 0.05) (Figure 2). These results indicated that knockdown of miR-210 decreased HIF-1α protein and increased protein expression of miR-210 targeted MNT, EFNA3 and AIFM3 genes in human hepatoma xenograft, which might lead to cell proliferation and angiogenesis suppression and apoptosis enhancement.

### Effect of knockdown of miR-210 in combination with radiotherapy on human hepatoma xenograft growth in athymic nude mice

**Figure 3:**
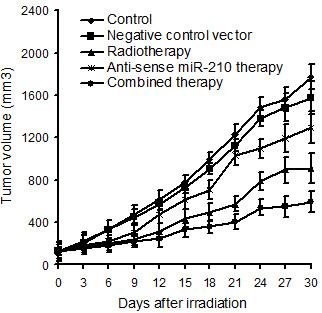
**Effect of knockdown of miR-210 in combination with radiotherapy on human hepatoma xenograft growth.** The tumor volume of five groups with 6 animals in each group is shown. The tumor volume was significantly decreased in mice of radiotherapy group (from day 6 to 30), anti-sense miR-210 therapy group (from day 6 to 30) and combined therapy group (from day 6 to 30) compared with control group. In addition, the tumor volume was significantly decreased in mice of combined therapy group compared with anti-sense miR-210 therapy (from day 9 to 30) or radiotherapy group (from day 12 to 30).

The tumors were irradiated with 8 Gy one day after dividing, followed by detection of hepatoma xenograft growth (Figure 3). It can be seen that the tumor volume was significantly decreased in mice of radiotherapy group (from day 6 to 30, *p* < 0.001), anti-sense miR-210 therapy group (from day 6 to 30, *p* < 0.01 or *p* < 0.001) and combined therapy group (from day 6 to 30, *p* < 0.001) compared with control group. The tumor volume in mice of negative control vector group showed no significant change compared with control group (from day 3 to 30, *p* > 0.05). The average tumor volume reached 1772 mm^3^ in mice of control group on day 30, while only 593 mm^3^ (33.46% of control) in combined therapy group, 1295 mm^3^ (73.10% of control) in anti-sense miR-210 therapy group, and 913 mm^3^ (51.52% of control) in radiotherapy group. In addition, the tumor volume was significantly decreased in mice of combined therapy group compared with anti-sense miR-210 therapy (from day 9 to 30, *p* < 0.01 or *p* < 0.001) or radiotherapy group (from day 12 to 30, *p* < 0.01 or *p* < 0.001).

### Effect of knockdown of miR-210 in combination with radiotherapy on survival of nude mice bearing human hepatoma

**Figure 4:**
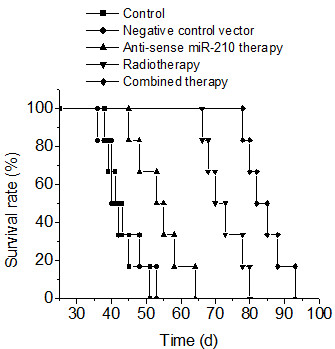
**Effect of knockdown of miR-210 in combination with radiotherapy on survival of mice bearing human hepatoma.** Results of survival were evaluated using Kaplan-Meier.

The long-term outcome of knockdown of miR-210 in combination with radiotherapy was evaluated by survival rates of mice bearing human hepatoma. Results of survival were evaluated using Kaplan-Meier (Figure 4). The median survival of control, negative control vector, radiotherapy, anti-sense miR-210 therapy and combined therapy group was 42.83 ± 4.75, 43.5 ± 6.06, 72.50 ± 5.58, 53.83 ± 6.85 and 84.33 ± 5.54 days, respectively. Survival durations were significantly longer in radiotherapy group (*p* < 0.001), anti-sense miR-210 therapy group (*p* < 0.001) and combined therapy group (*p* < 0.001) compared with control group. Survival durations were significantly longer in combined therapy group compared with radiotherapy (*p* < 0.01) or anti-sense miR-210 therapy group (*p* < 0.001).

### Effect of knockdown of miR-210 in combination with radiotherapy on cell proliferation in human hepatoma xenograft

**Figure 5:**
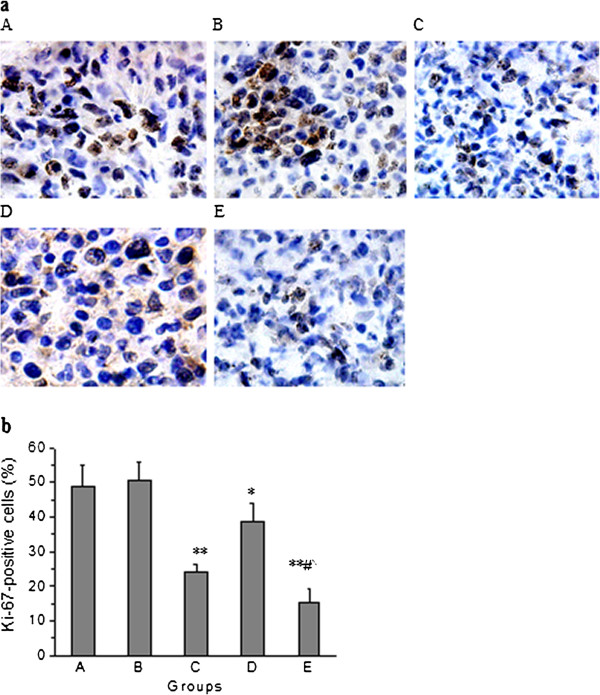
**Effect of knockdown of miR-210 in combination with radiotherapy on cell proliferation in human hepatoma xenograft.** Cell proliferation in human hepatoma xenograft was examined by Ki-67 staining. (**a**) Ki-67 immunohistochemical staining of tumor sections. **A**: control group; **B**: negative control vector group; **C**: radiotherapy group; **D**: anti-sense miR-210 therapy group; **E**: combined therapy group. The pictures are at 400× magnification. (**b**) Percentage of Ki-67-positive cells in stained tumor sections. Error bars indicate the standard error of the mean (SEM) (n = 9). * indicates *p* < 0.01, ** indicates *p* < 0.001 vs. control group; ^#^ indicates *p* < 0.001 vs. radiotherapy group; ^ indicates *p* < 0.001 vs. anti-sense miR-210 therapy group.

Effect of knockdown of miR-210 in combination with radiotherapy on cell proliferation in human hepatoma xenograft was examined by Ki-67 staining, which is a specific marker of proliferating cell. In microscopic observation of tumors, lesser number of Ki-67-positive cells was observed in radiotherapy group (*p* < 0.001), anti-sense miR-210 therapy group (*p* < 0.01) and combined therapy group (*p* < 0.001) compared with control group. The number of Ki-67-positive cells was significantly decreased in combined therapy group compared with radiotherapy group (*p* < 0.001) or anti-sense miR-210 therapy group (*p* < 0.001) (Figure 5).

### Effect of knockdown of miR-210 in combination with radiotherapy on angiogenesis in human hepatoma xenograft

**Figure 6:**
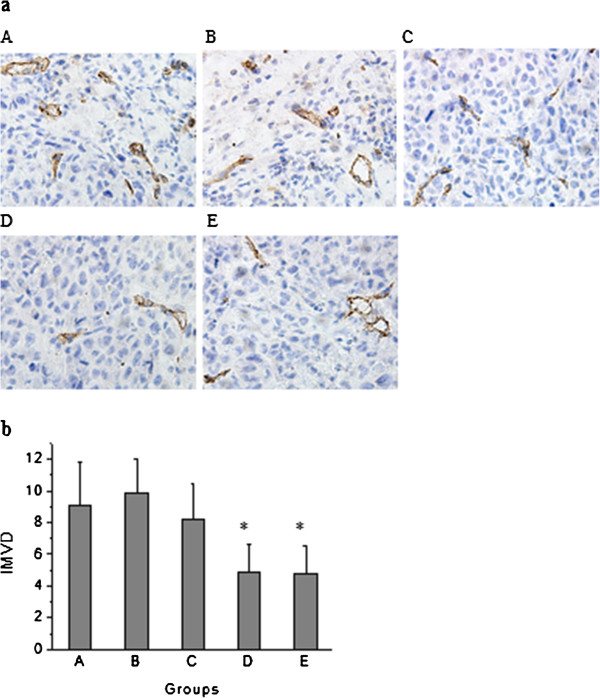
**Effect of knockdown of miR-210 in combination with radiotherapy on angiogenesis in human hepatoma xenograft.** Tumor angiogenesis was analyzed by CD31 staining. (**a**) CD31 immunohistochemical staining of tumor sections. **A**: control group; **B**: negative control vector group; **C**: radiotherapy group; **D**: anti-sense miR-210 therapy group; **E**: combined therapy group. The pictures are at 400× magnification. (**b**) IMVD in stained tumor sections. Error bars indicate SEM (n = 9). * indicates *p* < 0.01 vs. control group.

Effect of knockdown of miR-210 in combination with radiotherapy on tumor angiogenesis was analyzed by CD31 staining, which is a specific marker of endothelial cells. The pattern of staining was membranous and cytoplasmic. The microscopic examination revealed lower IMVD in treatment groups of tumors. The quantification of IMVD showed 9.11 ± 2.67, 9.89 ± 2.09, 8.22 ± 2.22, 4.89 ± 1.76 and 4.78 ± 1.79 in control, negative control vector, radiotherapy, anti-sense miR-210 therapy and combined therapy group, respectively. IMVD was significantly decreased in anti-sense miR-210 therapy group (*p* < 0.01) and combined therapy group (*p* < 0.01) compared with control group (Figure 6).

### Effect of knockdown of miR-210 in combination with radiotherapy on apoptosis in human hepatoma xenograft

**Figure 7:**
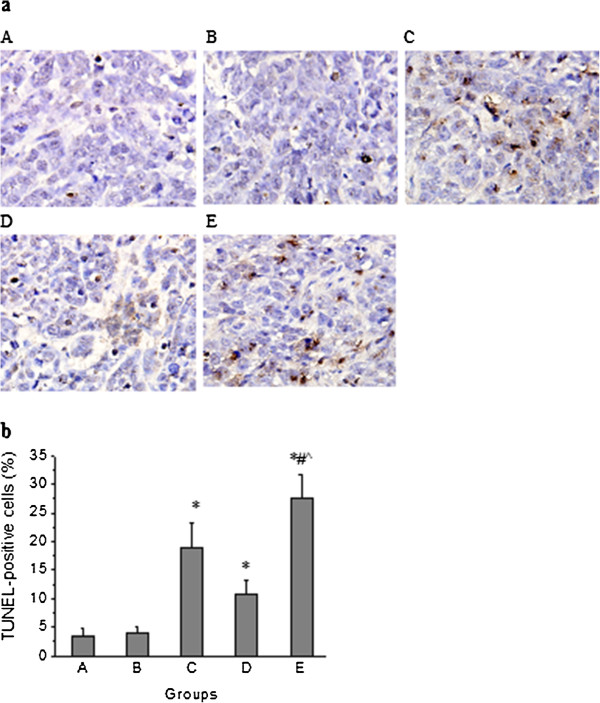
**Effect of knockdown of miR-210 in combination with radiotherapy on apoptosis in human hepatoma xenograft.** Apoptosis in tumors was analyzed by TUNEL staining. (**a**) TUNEL staining of tumor sections. **A**: control group; **B**: negative control vector group; **C**: radiotherapy group; **D**: anti-sense miR-210 therapy group; **E**: combined therapy group. The pictures are at 400× magnification. (**b**) Percentage of TUNEL-positive cells in stained tumor sections. Error bars indicate SEM (n = 9). * indicates *p* < 0.001 vs. control group; ^#^ indicates *p* < 0.001 vs. radiotherapy group; ^ indicates *p* < 0.001 vs. anti-sense miR-210 therapy group.

TUNEL staining was done to assess the apoptotic effect of knockdown of miR-210 in combination with radiotherapy in tumors, which showed an increased number of TUNEL-positive cells in treatment groups compared with control group. The quantification of TUNEL staining showed 3.44 ± 1.42%, 3.89 ± 1.05%, 18.89 ± 4.28%, 10.89 ± 2.42% and 27.67 ± 3.87% positive cells in control, negative control vector, radiotherapy, anti-sense miR-210 therapy and combined therapy group, respectively. TUNEL-positive cells was significantly increased in combined therapy group compared with radiotherapy group (*p* < 0.001) or anti-sense miR-210 therapy group (*p* < 0.001) (Figure 7).

## Discussion

The stem–loop of miR-210 is located in an intron of a noncoding RNA, which is transcribed from AK123483 on chromosome 11p15.5 [11]. MiR-210 is regulated by HIF-1α, HIF-2α and nuclear factor κB (NFκB) [11,13,20]. HIF-1α directly binds to a hypoxia responsive element (HRE) on the proximal miR-210 promoter, located 400 bp upstream of the structure [11]. NFκB p50 can physically interact with a conserved κB binding site and activate miR-210 promoter under hypoxia [20].

MiR-210 expression is elevated in a variety of human solid tumors [21,22]. The role of miR-210 in tumorigenesis has been investigated in several reports. However, the results of these experiments are somewhat controversial. It has been reported that high levels of miR-210 were associated with disease recurrence and short overall survival in head and neck squamous cell carcinoma [23] and display an inverse correlation with disease-free and overall patient survival in human breast cancer samples [24]. In addition, miR-210 levels correlate with breast cancer aggressiveness and metastatic potential [25]. However, genomic deletions of miR-210 in human epithelial ovarian cancer samples suggested these deletions as a possible trigger to tumorigenesis [12]. Huang *et al.* have demonstrated that stably expression of miR-210 in implanted tumor tissue could repress tumor growth in immunodeficient mice [26]. Our preliminary findings suggested that miR-210 might be a potential therapeutic target and specific inhibition of miR-210 expression in combination with radiotherapy showed an enhanced effect on hypoxic human hepatoma cells *in vitro*[17]. In the present study, in order to investigate the effect of knockdown of miR-210 in tumorigenesis in vivo, we subcutaneously implanted miR-210 downregulated human hepatoma SMMC-7721 cells into nude mice. We found that tumor growth was significantly delayed in SMMC-7721/Lv-anti-210 xenograft. To investigate the mechanism underlying the knockdown of miR-210 mediated tumor growth delay, we analyzed protein expression of HIF-1α gene and miR-210 targeted MNT, EFNA3 and AIFM3 genes in human hepatoma xenograft by Western blot. MNT represses Myc target genes by binding the E box DNA sequence (CANNTG) after forming heterodimers with Max [27,28]. MiR-210 could override hypoxia-induced cell cycle arrest by downregulating MNT [13]. EFNA3 is an ephrin family member involving vascular development [29]. Over-expression of EFNA3 significantly blocked the angiogenesis effect of miR-210 [30]. AIFM3, a gene homologous to the apoptosis-inducing factor (AIF), is a direct target of miR-210 in human hepatoma cells [17,31]. AIFM3 increases cytochrome *c* release and induces apoptosis in a caspase-dependent manner [32]. Our preliminary studies showed that AIFM3 downregulation by siRNA attenuated radiation induced apoptosis in miR-210 downregulated hypoxic human hepatoma cells, which suggest miR-210 downregulation mediate enhanced radiation induced apoptosis in hypoxic human hepatoma cells through AIFM3 gene at least in part [17]. The Western blot results indicated that knockdown of miR-210 decreased HIF-1α protein and increased protein expression of MNT, EFNA3 and AIFM3 genes in human hepatoma xenograft. HIF-1α protein downregulation by knockdown of miR-210 might be due to destruction of a hypoxia-induced positive feedback loop, in which HIF-1α induce miR-210 expression in hypoxia, which represses glycerol-3-phosphate dehydrogenase 1-like (GPD1L), contributing to increase of HIF-1α protein stability [33]. HIF-1α downregulation may inhibit proliferation, induce apoptosis, and enhance radiosensitivity in hypoxic cancer cells [4,5]. In immunohistochemical studies anti-sense miR-210 therapy group showed decreased Ki-67-positive cells and IMVD and increased TUNEL-positive cells compared with control and negative control vector group. These results suggest that knockdown of miR-210 may lead to tumor growth delay by cell proliferation and angiogenesis suppression and apoptosis enhancement.

In order to investigate the efficacy of knockdown of miR-210 in combination with radiotherapy in nude mice bearing human hepatoma SMMC-7721 cells, mice were monitored for tumor growth and survival after treatment as described in Materials and Methods. Results showed that the average tumor volume in combined therapy group reached 593 mm^3^ on day 30, only 33.46% of control group. In addition, survival durations were significantly longer in combined therapy group compared with control, radiotherapy or anti-sense miR-210 therapy group. These results suggest that knockdown of miR-210 in combination with radiotherapy is more effective than radiotherapy alone or anti-sense miR-210 therapy alone in suppressing tumor growth and extending survival duration. Analyzing associated mechanisms of the in vivo efficacy of combined therapy, we observed its inhibitory effects on cell proliferation (by Ki-67 staining) and tumor angiogenesis (by CD31 staining) and an enhancing effect on apoptosis (by TUNEL staining) in human hepatoma xenograft.

## Conclusions

In summary, our studies demonstrated that knockdown of miR-210 inhibited proliferation and angiogenesis, induced apoptosis in human hepatoma SMMC-7721 xenograft and knockdown of miR-210 in combination with radiotherapy showed an enhanced anti-tumor effect on human hepatoma. These findings suggest that specific inhibition of miR-210 expression may be a means to enhance the effectiveness of radiotherapy to human hepatoma.

## Competing interests

The authors declare that they have no competing interests.

## Authors’ contributions

WY conceived of the study design, performed all experiments and wrote the manuscript. JW helped to irradiate the tumor xenograft and measure the tumor diameters. TS participated in the conception of the study and interpretation of data. FJL performed critical revision of the manuscript. All authors read and approved the final manuscript.
